# Early-life contact with non-maternal adult cows and a pasture-based rearing environment influence behavioural responses of dairy heifers to novelty

**DOI:** 10.1017/awf.2024.20

**Published:** 2024-04-08

**Authors:** Laura Field, Lauren Hemsworth, Ellen Jongman, David McGill, Megan Verdon

**Affiliations:** 1 Animal Welfare Science Centre, Faculty of Science, University of Melbourne, Corner Flemington Road and Park Drive, Parkville, VIC 3052, Australia; 2 Institute of Future Farming Systems, School of Health, Medical and Applied Sciences, CQUniversity Australia, Rockhampton, QLD 4701, Australia; 3 Tasmanian Institute of Agriculture, University of Tasmania, Private Bag 3523, Burnie, TAS 7320, Australia

**Keywords:** animal welfare, calf development, environmental enrichment, novel object, pasture access, social enrichment

## Abstract

The complexity experienced in early life can affect trait development of individuals, including sociability and fearfulness. The modern dairy calf’s rearing environment often lacks environmental and social complexity. This study examined the effects of early-life, non-maternal adult contact and access to a physically complex environment on the responses of dairy heifers to several stressors, including restraint, social isolation, and novelty at 18 months of age. From the age of 2–13 weeks, 60 dairy heifers (*Bos taurus*) were reared according to one of three treatments applied to 20 calves each: (1) Hand-reared at pasture in groups of ten, with three unrelated dry cows (+S); (2) Hand-reared at pasture in groups of ten (–S); or (3) Hand-reared in sheds in groups of 10–12 as a commercial control (CC). At 13 weeks, all treatment groups were mixed and managed at pasture as a single herd. At 18 months, the responses of 50 heifers to restraint in a crush, social isolation and a novel object were observed (+S = 16, –S = 17, CC = 17). Treatment did not influence responses to restraint or social isolation, but influenced some indicators of fearfulness during exposure to a novel object. Six +S heifers interacted with the novel object compared to 0 –S and one CC, and CC heifers spent around 50% more time in vigilance than +S or –S heifers. Dairy heifers provided with early-life social enrichment in the form of non-maternal adult contact may have reduced fear of novelty. The implications for lifelong ability to adapt to novel situations, such as entry into the milking herd, should be assessed.

## Introduction

Dairy cattle (*Bos taurus*) experience social, cognitive, and physiological challenges throughout their lifecycle, often related to routine management procedures. Different physiological stages (e.g. pregnancy, parturition, lactation) are accompanied by changes to feeding regimes and daily routine, adaptation to the milking parlour, and associated regrouping. It is important that dairy cattle can adapt to management changes, novel environments, and changing social groups with as little stress as possible (for a discussion, see Gaillard *et al.* 2014). Animals who show lower levels of fear during routine events have been observed as having improved health, productivity, handling ease, and welfare (Breuer *et al.* 2000; Van Reenen *et al.* 2013; Hedlund & Løvlie 2015). Fearful animals, for instance, may be more difficult and dangerous for stockpeople to handle, reduce farm efficiency and damage infrastructure, increase stress for the wider group of animals, and may be culled more quickly; fearfulness may also indicate reduced welfare of the individual in question (Müller & Schrader 2005). Sustained and excessive stress can, furthermore, lead to chronic stress with negative welfare and productivity outcomes (Forkman *et al.* 2007). ‘Boldness’, meanwhile, has been correlated positively with growth rates of beef cattle, while dairy cattle deemed ‘fearful’ appear to have reduced milk production (Breuer *et al.* 2000; Biro & Stamps 2008; Hedlund & Løvlie 2015; Neave *et al.* 2022).

Behavioural tests conducted under research conditions (e.g. social isolation or novelty) provide insights into the behavioural responses expected from individuals when faced with similar stressors under commercial conditions. Where responses prove repeatable over time, consistent personality traits of individuals can be identified (see, for instance, Müller & Schrader 2005; Hedlund & Løvlie 2015; reviewed by Forkman *et al.* 2007). Adrenocortical responses and behavioural reactivity to novelty and social isolation in calves are consistent over time, and responses of both beef and dairy cattle to a novel object may predict future responses to risky or novel situations (Müller & Schrader 2005; Van Reenen *et al.* 2005; Kilgour *et al.* 2006; Takola *et al.* 2021).

Manipulating the individual’s early life experiences can permanently alter the way they interact with the world around them, including how they respond to stressful or challenging situations. For instance in social species, adult contact during early life, particularly with the mother, can reduce fearfulness (dairy cattle: Duve *et al.* 2012; Wagner *et al.* 2013; Meagher *et al.* 2015; Santo *et al.* 2020, chickens (*Gallus gallus domesticus*): Edgar *et al.* 2016, sheep (*Ovis aries*): Napolitano *et al.* 2008, quail (*Coturnix coturnix*): Pittet *et al.* 2014), and increase motivation to form and maintain social relationships (dairy cattle: Wagner *et al.* 2013, quail: Pittet *et al.* 2014), compared to animals reared without maternal contact. Animals reared with maternal contact also show improved competence in spatial or cognitive challenges (dairy cattle: Meagher *et al.* 2015, humans: Latham & Mason 2008, quail: Pittet *et al.* 2014). In dairy cattle, the effects of early-life adult contact, whether with the dam or with non-maternal adults, have been shown to last at least two years after cow-calf separation (e.g. Wagner *et al.* 2012, 2015; Field *et al.* 2023b). Wagner *et al.,* for instance, found that at 25 months of age, dairy heifers reared full-time with the dam and other mature cows were more submissive to mature cows when integrating into the milking herd (2012) and at 31 months had lower heart rates during isolation than heifers reared with twice-daily suckling dam contact or heifers reared artificially (2015). Field *et al.* (2023b), meanwhile, found that 23 month old heifers reared with non-maternal mature cow contact at pasture displayed similar grazing behaviour to mature cows within 24 h of first integrating into the main herd, while heifers reared at pasture or in sheds without adult contact did not.

Most research into calf early-life social experiences has explored the effects of maternal contact or individual vs pair- or group-housing of calves (see, for instance, De Paula Vieira *et al.* 2010; Wagner *et al.* 2015; or Buchli *et al.* 2017). Contact with non-maternal adults may provide an alternative to maternal adult contact where cow-calf contact is not feasible. Non-maternal adults could fill the role of social model and encourage the development of socially facilitated behaviours, without the management complications associated with managing lactating dairy cattle together with calves. Limited studies have explored the effects of exclusively non-maternal adult contact on the development of juveniles. Contact with older non-maternal animals appears to improve feeding behaviour in juvenile dairy cattle (Velázquez-Martínez *et al.* 2010; Costa *et al.* 2016) and increases positive social behaviour and reduces agonistic social interactions in juvenile horses (*Equus caballus*) (Bourjade *et al.* 2008). Field *et al.* (2023b) found dairy heifers that were reared with non-maternal adult cows had improved social capability and adaptability when mixed with adult cattle at 23 months, compared to heifers reared outdoors or indoors without adult contact.

Conventional indoor artificial rearing of dairy calves restricts access to adult contact as well as to the environmental complexity more readily available in naturalistic environments. In mice (*Mus musculus*), early-life environmental complexity improves social interaction strategies and reduces activity in an open field test, suggested by the authors to indicate a different way of responding to a novel environment, or that the early-life environment provided these individuals with more opportunities to hide or hold their own space (Pietropaolo *et al.* 2004). It also increases the likelihood of chickens to approach a novel object (Brantsæter *et al.* 2016). In dairy cattle, enrichments such as increased space allowance, full- or part-time access to the dam and other adults, or the presence of items such as brushes, chains or teats can be introduced to traditional indoor housing to improve environmental complexity, and may reduce fearfulness and lead to improved sociability and adaptability, daily weight gain and play behaviour (Jensen *et al.* 1998; Rushen & de Passillé 2014; Wagner *et al.* 2015; Zhang *et al.* 2022 and reviewed by Cantor *et al.* 2019). The specific effects of pasture-based housing on the long-term behavioural development of artificially reared calves, without the influence of adult contact, requires investigation, as research to date tends to centre on shorter-term behavioural, grazing or growth outcomes (e.g. Chambers 1959; Noller *et al.* 1959; Field *et al.* 2023a).

The present study explored whether rearing replacement dairy heifers with older, non-maternal adult dry cows from the ages of 2–12 weeks on pasture would affect long-term behavioural development. To test this, for their first three months of life, 60 dairy heifers were raised in one of three treatments differing in physical and social enrichment. At 18 months of age, 50 of the experimental heifers were exposed to restraint in a crush, a social isolation (SI) test and a novel object (NO) test. We hypothesised that heifers reared outdoors with mature dry cows as social companions would struggle less during restraint, be less active but more vocal during a period of social isolation, and interact with a novel object more quickly, more frequently and for a longer duration than heifers reared outdoors or indoors in groups of a single age with no mature cow contact.

## Materials and methods

This experiment was conducted at the Tasmanian Dairy Research Facility (TDRF, near Elliott in north-west Tasmania, Australia; 41°08′S, 145°77′E; 155 m above mean sea level) from August–November 2019. Behavioural testing was conducted in January 2021. All animal procedures were approved by the University of Tasmania Animal Ethics Committee (A0018141) under the Tasmanian Animal Welfare Act (1993). At the conclusion of behavioural testing, all animals were returned to the TDRF herd.

### Study animals

The 60 heifers studied were born within 35 days of each other during a concentrated peak of sexed semen calvings, and identically managed for their first two weeks of life. That is, all calves were born at pasture and separated from their dams within 12 h of birth. After being relocated from the calving paddock to woodchip-bedded, group-housing pens in a three-sided shed containing 12 calves each, they were bottle-fed colostrum twice within 24 h of birth (total 4 L), after which they were fed 2.5 L whole milk twice a day from fence-mounted 12-teat milk feeders. Calves had *ad libitum* access to water and calf starter concentrate pellets from birth. Calf starter concentrate was a blend of cereal grains, protein meals, legumes, vitamins, minerals and lucerne, formulated with minimum 20% crude protein, maximum 9% crude fibre and a minimum of 12.8 MJ ME kg DM^–1^. Access to these resources continued from birth to post-weaning when all heifers were returned to the research farm for commercial management.

### Treatment period

Management stages are outlined in Table 1. Full details of the early-life management protocol for +S and –S calves and the dry non-maternal cows are described in Field *et al.* (2023a). At 14–18 days of age (mean = 16.35 days), 40 of the 60 heifers, born within 12 days of each other during the herd’s first calving peak, were randomly allocated to one of four groups of ten animals, balanced for age, breed, and weight, for imposition of one of two treatments applied until 12 weeks (n = 2 groups per treatment): (1) Hand-reared, group-housed calves (–S); or (2) Hand-reared, group-housed calves housed with three non-familial dry cows (+S). Six mixed-breed multiparous dry dairy cows therefore participated in this initial experimental treatment period. The six available mature cows were paired according to similarity of traits (i.e. age, breed, weight), before random allocation of each member of the pair to one of the two +S groups (Replicate 1: 5 [± 1] years old, 516 [± 28.6] kg; Replicate 2: 6 [± 2.7] years old, 566 [± 69] kg). Replicate 1 cows therefore comprised one four year old Friesian, a five year old FJ-cross and a six year old FJ-cross. Replicate 2 cows comprised one four year old Friesian, one five year old FJ-cross, and one nine year old Friesian. Mature cow groups were introduced to their assigned groups of ten +S calves with 24 h fenceline contact before mixing, followed by an intensive 48 h observation period to ensure all animals transitioned into their mixed social groups appropriately.

**Table 1.** The housing and management of experimental heifers (+S, –S and CC) from birth until behavioural testing at 18 months

Age	0–2 weeks	2–13 Weeks	13 weeks–18 months	18 Months	18 months–calving
Experimental (+S) n = 20 over 2 replicates	Sheds	Experimental period – at pasture with cows	Mixed and housed at pasture	Behavioural testing +S n = 16 –S n = 17 CC n = 17	Mixed and housed at pasture
Experimental (–S) n = 20 over 2 replicates	Sheds	Experimental period – at pasture			
Commercial Control (CC) n = 20 over 2 replicates	Sheds	Retained in sheds			

In brief, each +S and –S group was housed on approximately 0.5 ha of cultivated ryegrass pasture for the duration of the treatment period and offered 3 L whole milk twice daily from fence-mounted ten-teat milk feeders. Within replicates, the +S treatment group was housed on one half of a 1 ha paddock, with the replicate’s respective –S group housed on the other half (for visual depiction of paddock layout, see Field *et al.* 2023a). Visual barriers were constructed from doubled hessian at 0.8–1.0 m height, and buffer zones of 15–20 m were constructed using four-string electrical tape, providing visual and physical but not audial barriers between the two treatment groups per replicate. A large pine hedge separated the two replicates and provided a windbreak.

All paddocks contained a water trough, and a creep area approximately one quarter of the length of the paddock and accessible only by calves, containing a three-sided shelter measuring approximately 2 × 2.5 m (length × width) in which concentrate was provided. The health and welfare of cows and calves were assessed by the first author (LF) twice daily. A single calf required treatment for bloat and unusual stool during the treatment period, which resolved within 24 h.

Of the 60 heifers enrolled in this study, the final 20 were born on the nearest days immediately preceding and following the births of calves allocated to –S and +S treatments. These calves formed two replicates of a commercial control (CC) group, which were managed from birth until weaning in groups (group size reduced to ten calves per pen), housed in bedded pens measuring approximately 32 m^2^ (stocking density 3.2 m^2^ per calf) and fed and monitored twice daily by the TDRF professional calf-rearing team. These calves were fed 5 L whole milk daily from fence-mounted 12-teat milk feeders until gradual weaning over 5–7 days at 12 weeks as per the research farm’s commercial practice. Discrepancies between quantity of milk offered to pasture- and shed-reared calves arise from differences between TDRF commercial practice and the experimental design, which aimed to provide sufficient nutritional support to calves housed outdoors. Wood-chip bedding was topped up when soiled but otherwise remained undisturbed during the milk-feeding period, and grass hay was provided daily. These heifers were recruited to the study at 18 months of age.

The TDRF herd is comprised several dairy breeds and all experimental animals retained by the farm to testing were Friesian, Jersey, or their associated crosses (Friesian +S = 10, –S = 9, CC = 6; Jersey +S = 1, –S = 2, CC = 1; FJ, FFFJ, FJJJ or majority FJ × other dairy genetics +S = 5, –S = 6, CC = 10).

At 12 weeks, mature dry cows were removed from +S paddocks, and all experimental calves were gradually weaned over the following seven days, remaining in their respective treatment groups. +S and –S heifers remained at pasture with continued access to concentrate and fresh water. Gradual weaning was achieved by reducing the amount of milk offered at afternoon feed by 0.5 L per day for four days, then removing the afternoon feed and reducing the milk offered at morning feed by 0.5 L per day for three days. Once daily milk allowance was reduced to 1.5 L, milk was no longer offered. At the completion of weaning, the youngest experimental calf was 13 weeks old. Heifers of all treatments were then mixed together into the larger replacement heifer herd at pasture at approximately 13 weeks of age. Heifers were managed identically at pasture with some provision of supplementary forage and checked daily and weighed monthly by farm staff, as per typical management of a dairy heifer in a pasture-based system (see Verdon 2023).

### Animal management between treatment and behavioural testing

Heifers were submitted to the farm breeding programme at 13 months of age. After pregnancy testing, 50 of the 60 experimental heifers remained on-farm to participate in behaviour testing at 18 months (+S = 16, –S = 17, CC = 17). Testing was conducted at 18 months to work within farm management constraints while also examining the longitudinal effects of early-life treatment. Of the ten heifers that were not retained, all were sold by the farm: three did not meet the farm’s genetics programme protocol, and seven were not in calf.

### Restraint scoring: Pre-test procedure

At 18 months, the 50 experimental heifers remaining in the farm’s replacement herd were drafted from the larger herd of pregnant heifers for behavioural testing. On the day of restraint scoring the replacement heifer herd, including these 50 heifers, were walked together from their paddock to a large set of grassed yards fitted with a water trough. Heifers were regularly handled in these yards, including being weighed monthly in the crush connected to a race adjacent to the yards from 3–18 months of age. +S and –S heifers, but not CC heifers, had also been weighed in the yard-adjacent race weekly from 8–12 weeks of age. After 30 min of acclimatisation to the stockyards in the larger group, each animal was moved through the yards in turn, up the race and into the steel crush; experimental animals were therefore scored in the order in which they entered the race and came through the crush.

### Restraint scoring: Test procedure

Each heifer was calmly and gently walked up the race and into the crush in turn by two researchers. Once all four of the heifer’s hooves had entered the crush, the back gate was closed, and observations commenced as soon as the gate was shut. A single researcher standing within 2 m to the left side of the heifer, in line with the back gate, continually observed and scored the behavioural responses of the heifer to restraint for 20 s, using a five-point subjective scale score (for full scoring system, please refer to Lees *et al.* 2020). The scale ranged from a score of 1 (heifer calm, standing still, head mostly still, slow calm movements) to 5 (heifer very nervous, violent movements, rearing, attempting to jump out). The other researcher remained out of proximity of the crush. At no point was a head bale or squeeze employed, and all researchers remained motionless and silent for this time. The crush test process took approximately 120 min in total, including weighing non-experimental heifers.

### Behavioural testing: Pre-test procedure

Immediately following restraint scoring, the weight of each heifer was recorded before the heifer was individually marked on both sides using coloured stock spray and randomly allocated into one of four groups, balanced for treatment, to be tested on each of the four consecutive testing days. Testing day was then randomly allocated per group. Twelve heifers (+S = 4, –S = 4, CC = 4) were allocated for testing on day 1, while 15 (+S = 5, –S = 5, CC = 5), 12 (+S = 4, –S = 4, CC = 4) and eleven (+S = 3, –S = 4, CC = 4) were allocated to the subsequent days. Two non-experimental heifers were randomly allocated to each group and remained with these groups for the duration of testing to ensure that no animals would be housed alone at any point. Heifer groups were left to stabilise for four days prior to behaviour testing, with each group housed separately in adjacent nearby paddocks, where fresh water and pasture were available at all times. Behavioural testing in the arena took place over four days, with one group tested per day. Throughout the testing period, groups not participating in testing remained in their paddocks.

### Behavioural testing: Testing arena

**Figure 1.**
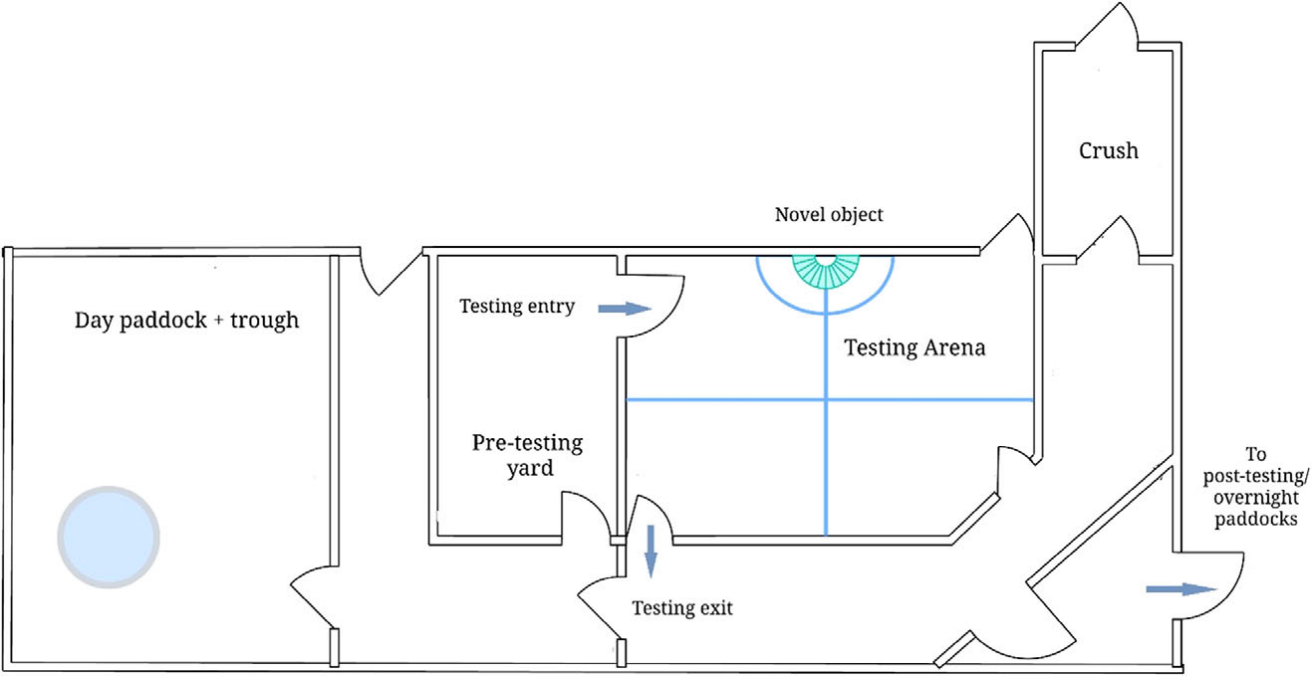
The layout of the testing environment, constructed around existing stockyards. This includes the crush used in restraint scoring, and the testing arena used for social isolation and novel object testing. The locations of day and night paddocks are also indicated (not to scale).

The behavioural testing arena (approximately 9 × 9 m; Figure 1) was erected within a grassed central area of the pre-existing stockyards. Plywood was attached to the interior of the arena to a height of 1.8 m. A hole was drilled into one side of the arena to allow novel object entry. The novel object was a large rainbow umbrella (Shelta, Sydney, Australia; 90 × 104 cm; length × depth) which was inserted, unopened, through the hole, and then slowly opened and pulled against the arena wall once open. The novel object entry hole was sealed using a block of wood when not in use. The arena floor was marked into quarters using stock spray. A semi-circle 1 m in radius was marked on the arena floor and up the walls on either side of the novel object entry hole (Figure 1). Five cameras (Go-Pro Hero7, GoPro Inc, San Mateo, CA,USA) were attached to the walls of the arena and continuously recorded its interior from all angles. Heifers within the arena did not have physical or visual contact with other animals, but audial contact with the larger herd could be maintained.

### Behavioural testing: Test procedure

Differences in weather across the four testing days, collected from data available at the Australian Bureau of Meteorology at the time of testing, are presented in Table 2.

**Table 2.** Weather data for each of the four consecutive testing days, obtained from records available from the Australian Bureau of Meteorology

Testing day	Max temp (°C)	Wind	Cloud	Precipitation
1	29	None	Passing	Light drizzle
2	22	Windy	None	None
3	22	Light	Passing	None
4	18	Very windy	None	None

Immediately prior to testing, the group of heifers being tested was walked from their paddocks to the testing arena. The entry gate to the arena was closed and the heifers were given 10 min to explore and acclimatise to the arena as a group before the exit gate was opened and heifers were walked to the holding paddock. Testing commenced immediately following this acclimatisation period.

Heifers were tested individually between 0920 and 1500h. Treatment group of the first animal tested per testing day was randomised between days. Tests were conducted in sequences of three (one animal per treatment), with treatment order of testing randomised within days. This meant that one animal from each treatment was tested, followed by a second animal from the next treatment, followed by a third from the third treatment, and so on until all the animals and treatments had been tested. When not being tested, heifers were contained in a large grass holding yard near the testing arena. Water was available *ad libitum*, while fresh allocations of grass were provided within the holding yard.

Individual heifers were brought to the test area, the entry gate was closed, and the heifer was left alone for 7 min. The novel object was then inserted through the hole and opened, then held in this position for a further 7 min before the exit gate was opened and the heifer gently ushered from the arena to return to her overnight paddock. These times were chosen with reference to Buchli *et al.* (2017) who used a 5-min acclimatisation period as a social isolation test before introducing a novel object to the arena for 5 min, and a review of fear tests in cattle by Forkman *et al.* (2007) who indicate that cattle are generally given between 1 and 15 min to habituate to a novel arena prior to the introduction of the novel object. The two non-experimental heifers housed with each group remained in the holding yard until all experimental heifers had been tested; this ensured that no animals were housed alone in the holding yard prior to testing.

### Data collection: Behaviour

Video recordings were analysed continuously by a single, trained observer using Behavioural Observation Research Interactive Software (BORIS; Friard & Gamba 2016). Behaviours recorded are outlined in Table 3. Intra-observer reliability for behavioural test observations was undertaken on total duration and frequencies of behaviours from a random sample of 6% of tests (50% of two tests per treatment for each of the isolation and novel object tests). Intra-observer reliability of frequency observations calculated using Cohen’s kappa was substantial (κ = 0.630; *P* = 0.000), and of duration observations calculated using Spearman’s rho was strongly correlated at 0.997 (*P* = 0.000). The observer was familiar with the animals and may have been aware of treatments imposed on certain individuals; the observer could not be blinded to treatment beyond the use of number/colour codes during testing and while data were collected during behaviour test observations.

**Table 3.** Ethogram of behaviours recorded for social isolation and novel object tests^a^

Behaviour	Description	**Test^b^**	Measure
Vigilance	While standing stationary. Head position above horizontal to the withers, ears upright. A bout of vigilance may be interrupted, but for no more than 2 s.	SI, NO	Duration
Standing	Standing stationary, head position horizontal or at an angle below the horizontal to the withers or above the horizontal to the withers but without ears upright. May include calmly looking around environment, a bout of standing may be interrupted, but for no more than 2 s.	SI, NO	Duration
Self–grooming	Licking, rubbing and/or scratching own body.	SI	Duration
Grazing	Heifer lowers her head and does not only sniff the ground but also takes a bite of grass. She may continue to browse.	SI, NO	Duration
Walking	Walking more than two steps with forelegs, forwards or backwards.	SI, NO	Duration
Running	Trotting, cantering, or galloping more than two steps, including locomotor play (i.e. gallop with, or: leap, jump, buck, turn, kick out).	SI, NO	Duration
Explore environment	Interacting with (i.e. sniffing, licking, touching, chewing, rubbing) – but NOT forcefully pushing against – any part of the environment including the ground while standing or walking; not including grazing, for > 2 s. A bout can be interrupted, but not for more than > 2 s. For social isolation test, this does not include exploring gateways, for novel object this does include gateways.	SI, NO	Duration
Explore gateway	Interacting with any gateway including sniffing or licking, rubbing head, pushing nose gently through gate. Includes standing with contact, or with at least two hooves within 1 m of the gate with head orientated towards gate or standing parallel to the gate. A bout can be interrupted by another behaviour, but not for more than > 2 s. (Social isolation only).	SI	Duration
Attempt escape	Pushing head (beyond nose) through arena perimeter with force, jumping or pushing against or actively trying to get through arena perimeter, using head and/or body to actively bend arena materials.	SI, NO	Frequency
Elimination	Defaecation and/or urination	SI, NO	Frequency
Vocalisation	Any vocalisation by the heifer	SI, NO	Frequency
Behaviour transitions	Number (count) of times the heifer transitions between state behaviours (measured by duration) across the length of each respective test.	SI, NO	Frequency
Interact with novel object	The heifer makes physical contact with the novel object with her nose, mouth, or forehead, including licking and biting the novel object, sniffs the novel object and/or the distance between muzzle and object is no more than the width of the muzzle.	NO	Duration
Within 1 m of object	Any part of the heifer’s body is within 1 m of the novel object, as measured by 1 m proximity marks on walls and floor of arena.	NO	Duration

a Developed with reference to Van Reenen *et al.* (2013), Wagner *et al.* (2013); Hedlund & Løvlie (2015), Meagher *et al.* (2015) and Buchli *et al.* (2017).

b SI = Social Isolation; NO = Novel Object

Isolation (SI) observations commenced as soon as the testing arena gate was closed and finished after 7 min. Novel object (NO) observations ran for 7 min, commencing as soon as the tip of the umbrella passed through the novel object entry hole. Behaviours recorded during the isolation test include total time (s) spent in immobile vigilance, standing, self-grooming, walking, running, exploring the environment, and the total number of behavioural transitions (Table 3). Behaviours recorded during the novel object tests include those previously listed, as well as total durations of interactions with the novel object.

The novel object test video of one +S heifer could not be observed due to a technical malfunction. Data from this heifer were not included in NO analysis.

### Statistical analysis

#### Crush score

Crush score data were analysed with IBM® SPSS Statistics for Windows (IBM Corporation 2020, Version 27) using a Kruskal-Wallis test to explore whether there were significant differences between treatments. A non-parametric test was chosen to compare treatment differences as the single-score data were skewed towards the lower end of the five-point scale.

#### Social isolation and novel object tests

Behavioural observation data were exported from BORIS (Friard & Gamba 2016) at the level of total frequency and duration of each behavioural bout per behaviour. Total duration of the sum of all behaviours for each heifer was checked to ensure total observation duration was 420 s per behavioural test (± 5 s). All data met these criteria.

All behaviour test data were analysed in R Version 4.3.1 (R Core Team 2023). In the following descriptions of the statistical analysis, ‘Heifer’ denotes the individual animal from whom data were collected. ‘Treatment’ denotes +S, –S or CC management protocol. ‘Rearing group’ was included in all statistical models as a random effect to account for the individual group a heifer was reared with (i.e. group 1–6 during rearing, wherein +S Replicate 1 heifers belonged to Group 1, +S Replicate 2 heifers belonged to group 4, and so on). ‘Testing day’ was included in all statistical models to account for differences across the four consecutive days upon which testing took place.

Count data for eliminations, vocalisations and escape attempts were unsuitable for analysis and not explored further. Only a small number of heifers entered within 1 m of or interacted with the novel object (seven of the 49 heifers tested). Duration of interactions, number of interactions and proportion of heifers to interact with the novel object per treatment were correlated. Rather than calculating differences in latency to interact or duration of time spent interacting with or within 1 m of the novel object, a Pearson Chi-squared test was chosen to compare treatments for the proportion of animals of each treatment which interacted with the novel object within the 7-min test.

The total duration (s) of state behaviours as defined in the ethogram were aggregated at the heifer level for analysis per test, as was the total frequency that each heifer changed behaviour over the course of each behaviour test. Linear mixed effects models were fitted to behavioural test data assessing the total duration of time each heifer spent standing, walking, in vigilance, exploring the environment (and exploring the gateway in the social isolation test only), grazing and running, separately. The lmer() linear mixed model function was used from the ‘lme4’ package (Version 1.1) in R Version 4.3.1 ("Beagle Scouts"© R Core Team 2023) to fit all models. Treatment and Testing Day were included as fixed effects, while rearing group was included as a random effect. While heifer was considered as a random effect, due to the number of records within each grouping it was not included in the final model. The equation used in R was:(#Equation 1)

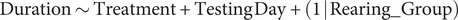

For frequency of behavioural transitions, data were analysed using a glmer() function to fit a generalised linear mixed-effects model function using a ‘Poisson’ distribution. Significance was determined at *P* ≤ 0.05 using anova() function to compare between models with and without the ‘Treatment’ effect.

Results from overall tests for effects of day on behaviours recorded are presented in the Supplementary material.

## Results

### Crush score

There were no differences in crush scores between treatment groups (*P* = 0.225). Scores above 3 were rarely observed and the median score for all groups was 2. CC heifers had a mean score of 2 (range 1–3), –S was 1.94 (range 1–4), and +S heifers 1.56 (range 1–3).

### Isolation test

Effects of treatment on duration of behaviours observed during social isolation are presented in Table 4 along with estimates and standard errors. No treatment effects on behaviours were found. Treatment also did not affect frequency of behavioural transitions across the duration of the isolation test (*P* = 0.281).

**Table 4.** Effects of treatment (+S, –S, and CC) on duration (s; ± SE) of behaviours observed during a 7-min social isolation test of dairy heifers

Behaviour	Estimate (± SE)			F (df)	*P-*value
	+S	–S	CC		
Explore environment (s)	58.0 (± 7.3)	58.6 (± 7.1)	63.6 (± 5.1)	1.115 (2, 44)	0.337
Explore gateway (s)	62.7 (± 8.5)	60.2 (± 8.3)	65.9 (± 6.0)	0.586 (2, 3.03)	0.609
Walking (s)	100.3 (± 6.9)	90.8 (± 6.8)	99.6 (± 4.8)	1.074 (2, 44)	0.350
Running (s)	3.4 (± 1.9)	5.1 (± 1.9)	5.9 (± 1.3)	1.710 (2, 43)	0.193
Vigilance (s)	21.0 (± 6.0)	38.6 (± 5.9)	31.8 (± 4.2)	1.661 (2, 44)	0.202
Standing (s)	123.1 (± 11.9)	89.7 (± 11.7)	99.5 (± 8.4)	1.989 (2, 44)	0.149
Grazing (s)	52.4 (± 13.5)	74.3 (± 13.3)	52.3 (± 9.5)	1.867 (2, 44)	0.167
Self–grooming (s)	2.3 (± 0.7)	2.9 (± 0.7)	2.5 (± 0.5)	0.149 (2, 44)	0.862

Values calculated with linear mixed effects models (#Equation 1). CC = heifers reared commercially in sheds, without adult contact; +S = heifers reared at pasture with adult contact and –S = heifers reared at pasture without adult contact

### Novel object test

Treatment did not affect the frequency of behavioural transitions across the duration of the novel object test (*P* = 0.239). Very few animals interacted with the novel object. Of the 49 novel object tests observed, six of the 15 +S heifers, 0 of the 17 –S heifers and one of the 17 CC heifers interacted with the novel object, with treatment effect on proportion of heifers to interact with the object confirmed in the Chi-squared test (*P* = 0.001).

Effects of treatment on duration of behaviours observed during the novel object test are presented in Table 5 along with estimates and standard errors. Only duration of time spent in vigilance was affected by treatment (*P* = 0.01). Pair-wise contrasts did not elucidate specific treatment differences, however +S and –S estimates for vigilance durations were almost identical, while CC heifers were estimated to spend approximately 50% more time in vigilance than +S or –S heifers. Testing day did not affect vigilance behaviour (*P* = 0.201).

**Table 5.** Estimates for effects of treatment (+S, –S, and CC) on duration (s; ±SE) on behaviours observed during a 7-min novel object test of dairy heifers

Behaviour	Estimate (± SE)			F (df)	*P-*value
	+S	–S	CC		
Explore environment	60.8 (± 12.0)	56.5 (± 11.6)	59.8 (± 8.3)	0.043 (2, 43)	0.958
Walking	45.5 (± 7.2)	39.7 (± 7.0)	41.3 (± 5.0)	0.176 (2, 43)	0.839
Running	0.2 (± 1.0)	3.5 (± 0.9)	1.9 (± 0.7)	1.997 (2, 43)	0.148
Vigilance	20.9 (± 5.5)	22.0 (± 5.4)	30.8 (± 3.9)	5.524 (2, 43)	0.007*
Standing	250.7 (± 29.2)	212.8 (± 28.7)	230.8 (± 20.5)	0.285 (2, 3.07)	0.770
Grazing	28.3 (± 22.6)	85.6 (± 22.3)	50.1 (± 15.9)	1.279 (2, 3.083)	0.394

Values calculated with linear mixed effects models (#Equation 1). CC = heifers reared commercially in sheds, without adult contact; +S = heifers reared at pasture with adult contact and –S = heifers reared at pasture without adult contact. Significant values are denoted by an asterisk and bold type.

### Effect of day

In the social isolation test, test day affected the duration of walking (*P* = 0.047), running (*P* = 0.045), vigilance (*P* = 0.004) and grazing (*P* = 0.000) behaviours. In the novel object test, test day affected the duration of walking (*P* = 0.023), standing (*P* = 0.005) and grazing (*P* = 0.001) behaviours. The inclusion of test day in the model accounted for these differences in the final model (#Equation 1).

## Discussion

This study examined the effects of early-life non-maternal adult contact and access to a more physically complex rearing environment on the longitudinal responses of dairy heifers to a series of stressors, including restraint, social isolation, and novelty. Treatments were imposed on heifers from 2–13 weeks of age, and behavioural testing took place when heifers were 18 months of age. Contrary to our hypotheses, there were no effects of early-life treatment on response to restraint in a crush or social isolation. As hypothesised, however, heifers reared at pasture in contact with non-maternal adults (+S) appeared to be less fearful in the presence of a novel object than heifers of other treatments. Heifers reared at pasture without adult contact (–S), in turn, appeared to be less fearful of novelty than heifers reared in sheds (CC).

Only a small number of behaviours were influenced by treatment in the novel object test: a higher proportion of +S heifers interacted with the novel object than –S or CC heifers, and +S and –S heifers were less vigilant than CC heifers when presented with the novel object. These behaviours can primarily be interpreted as indicators of fearfulness, defined in cattle as “a tendency to express fear when exposed to potentially threatening stimuli or situations” (Meagher *et al.* 2016). Increased complexity of the early-life social environment has previously been associated with evidence of reduced fearfulness. Access to a complex social group containing the dam, other adults and other calves reduces the initial cardiac response of calves in response to isolation compared to calves reared in same-age groups, and the latency of calves to approach a novel object compared to calves reared individually (Meagher *et al.* 2015; Buchli *et al.* 2017). Further, when compared to pair-housed calves, individually housed calves run and defaecate more often, explore less, have higher heart rates and are more reluctant to enter an unfamiliar arena, suggesting greater anxiety in these animals in response to a novel environment (Jensen *et al.* 1997; De Paula Vieira *et al.* 2012; Jensen & Larsen 2014; Lindner *et al.* 2024). The results of the present study provide further evidence that the complexity of the early-life environment may influence the long-term development of fearfulness in dairy heifers.

### Responses to novelty

In contrast with previous studies, only seven of the 49 18 month old experimental heifers approached and interacted with the novel object in the present study. Wagner *et al.* (2015) observed up to 24 of their 26 post-partum experimental animals approaching a traffic cone in the indoor walkway between the milking parlour and their home barn, for instance, while at the other end of the age spectrum an umbrella at the side of their outdoor rearing pens was approached by 43 out of 58 six week old calves studied by Mahendran *et al.* (2021). While in the present study each experimental treatment comprised a variety of dairy breeds, Mahendran *et al.* (2021) recruited only Holsteins and Wagner *et al.* (2015) studied both German Holsteins and German Red Pieds, with no reports of breed differences in behaviour in the latter study. The size of the arena in the present study was greater than in these cited studies; this combined with the location of the umbrella on the side rather than centre of the large arena may have reduced the motivation of heifers to enter its proximity.

Nevertheless, more +S heifers interacted with the novel object than –S or CC treatment groups. Wagner *et al.* (2015) similarly found calves reared without dam contact to be less likely to interact with a novel object compared to dam-reared calves. Exploratory behaviour is often associated with reduced fearfulness, and both are aroused by novelty. Hogan (2004) describes fear/exploration as a “unitary system that is expressed as approach at low levels, withdrawal at moderate levels and immobility at high levels”. Van Reenen *et al.* (2005, 2009) found that the time calves spend in contact with a novel object correlates negatively with cortisol responses and positively with increasing dosages of the anxiolytic drug, brotizolam. As exploration tends to increase as fearfulness decreases, we suggest that –S and CC heifers were generally more fearful than +S heifers when presented with novel stimuli in a familiar environment.

CC heifers also exhibited approximately 50% higher durations of vigilance during the novel object test compared to both the +S and –S heifers. These differences in vigilance were not observed in the social isolation test conducted immediately prior to the introduction of the novel object. As the immobile vigilance of CC heifers increased immediately following the introduction of the novel object, we suggest that this behaviour is directly linked to its presence. Vigilance has been associated with fearfulness or anxiety, unfamiliarity, unpredictability, or environments where the animal is deprived of the safety of the herd, with an associated increased potential for threats (Welp *et al.* 2004; Forkman *et al.* 2007). A greater tendency for CC heifers to exhibit immobile vigilance when faced with an unfamiliar object suggests increased fearfulness in these animals compared to both the +S and –S heifers.

Rather than being an indicator of overt fear, failure to interact with a novel object could suggest that an individual has reached an equilibrium of motivations in an approach-avoid conflict. In such circumstance, other behavioural indicators, such as the heightened vigilance of CC heifers, can help elucidate the level of fearfulness experienced by animals in the test (Hogan 2004). While –S heifers showed no inclination to explore the novel object, they also displayed no overt indicators of fearfulness, such as increased vigilance. Where testing protocol provides no external motivation to approach, an indifferent animal may be as unlikely to interact with a novel object as a fearful animal (Forkman *et al.* 2007). In the present study, the novel object was placed in a neutral location within a familiar environment; the only novel stimuli in an environment which provided no further motivation for heifers to interact with it than their own innate motivation to explore (Takola *et al.* 2021).

When describing the fear/exploration motivation system, Hogan (2004) defines three types of response: low fearfulness, characterised by a motivation to explore novelty (as demonstrated by the +S heifers); high fearfulness, characterised by a ‘freeze’ response (much akin to the immobile vigilance response of the CC heifers); and a moderate level of fearfulness characterised by a tendency to withdraw from novel stimuli. While –S heifers did not actively withdraw from the object, compared to +S and CC heifers their responses indicate moderate but not high fearfulness, primarily due to their failure to approach or interact with the novel object. Indeed, the +S and –S heifers spent an almost identical time displaying vigilance. Environmentally enriching the environment of calves housed indoors reduces their reactivity to novelty, increases rates of play and improves cognition (for a review, see Verdon 2021). We hypothesise that –S heifers experienced moderate fearfulness in the presence of the novel object, relative to the other two treatment groups, which appeared to experience low (+S) and high (CC) fearfulness, respectively. Physiological measures of fearfulness, including HPA responses, would be required to confirm this.

The trend for reduced fearfulness in response to novelty in both groups of pasture-reared heifers compared to CC heifers in this study suggests that both environmental and social enrichment affect the development of fearfulness in calves. In adult mice, both environmental (enriched cage) and social (pair housing vs isolation) enrichment during early life reduces movement in an open field test, but the combination of environmental plus social enrichment had the most profound effect on inactivity (Pietropaolo *et al.* 2004). In the present study, a combination of environmental and social enrichment during early life (+S) appeared to have the greatest effect in reducing fearfulness in response to novelty. Environmental enrichment, but not pair vs individual housing, improve memory and adaptability to change, and may also increase calf inclination to explore a novel object (Zhang *et al.* 2022). Future research with a factorial design may help to determine whether the effects of environmental and social enrichment on the development of neophobia in this study were cumulative or discrete.

### Response to social isolation

Social isolation is one of the greatest stressors sociable herd animals such as cattle can face, and social isolation tests are associated with increased cortisol concentration and behavioural reactions in dairy cattle (Müller & Schrader 2005). All the animals in the present study were equally familiar with the testing environment, including the location of entrances and exits. Social separation was therefore likely to have been the greatest stressor presented in the social isolation test.

The present study recorded no treatment differences in any behaviours during social isolation. These results contrast with those of Wagner *et al.* (2015) who found that dam-reared dairy cattle housed indoors during early life were more active during social isolation than artificially reared dairy cattle at approximately 31 months, while Le Neindre (1989) recorded similar results for foster-reared calves at 3.5 and 4.5 years of age compared to calves reared in isolation. This may be attributed to differences in the nature of early-life social contact and housing between these and the present studies; no calves in the present study were housed individually during early life, nor were they reared by their dams or allowed to suckle the non-maternal adults they were housed with, and adult contact was provided only to calves housed outdoors.

### Response to restraint

No treatment effects were observed in crush scores during 20 s restraint, and scores above 3 were rarely recorded. Grandin (1993) observed consistency in crush scores of individual beef cattle over time and concluded that extreme responses to restraint characterised by agitation in a crush were indicative of a poor temperament in the individual. Response to restraint in a crush was also used by Neave *et al.* (2022) as a measure of reactivity amongst five measures used to determine personality traits in dairy cattle. They found it to be highly repeatable and linked investigative behaviour of novelty with low reactivity to restraint (Neave *et al.* 2022). In contrast, Lee *et al.* (2018) explored differences in responses of Angus heifers treated with or without a drug to pharmacologically induce an anxiety-like state and suggest crush score does not suitably measure or indicate fearfulness in the individual. The heifers studied in the present research were well-handled dairy animals familiar with the crush used. Unlike the cows studied by Neave *et al.* (2022), they were also not restrained with a head bail or squeeze chute, or for the full 30 s as in previous studies (e.g. Lee *et al.* 2018). It therefore appears unlikely that our crush test was long, aversive, or stressful enough to elicit any agitation or other notable response in any of the animals.

### Effect of testing day

Testing day influenced walking and grazing behaviour in both the social isolation and novel object tests, running and vigilance behaviour in the social isolation test and standing behaviour in the novel object test. Few recorded factors seem likely to have affected behaviour across days (e.g. time of feeding and thus rumen fill at testing was comparable, no external stimuli such as tractors or moving cattle nearby were recorded). Behavioural differences across days may thus best be explained by differences in weather on each testing day, including temperature and wind strength (see Table 2). Such differences are less often experienced during tests conducted indoors and may account for some variability in results within treatments. Higher winds on days 2 and 4 of testing may explain relative inhibition of grazing behaviour on these days, for instance, due to a greater reticence to lower the head to a more vulnerable grazing position in these conditions. Vigilance behaviour during the novel object test was not affected by day, meanwhile, suggesting treatment effects for this behaviour withstood differences in testing conditions across days.

### Experimental limitations

The experimental design of the present study was restricted by certain limitations. Primarily, a greater sample size utilising more animals across more replications may have yielded stronger or more descriptive results. Pasture-based dairy systems are, however, characterised by large animals which must be housed appropriately and grazed rotationally in large-scale systems, and the logistical difficulties involved with replicating behavioural research under such conditions, which has been widely documented, were limiting factors in the present study’s experimental design (e.g. Phillips 2000; Oksanen 2001; Davies & Gray 2015; Bello *et al.* 2016; discussed in Field *et al.* 2023a). Where viable, future research may consider conducting similar experiments with replication over several years and calving seasons to meet similar levels of replication to those experiments conducted in intensive indoor systems.

The TDRF research herd comprises a mixture of popular Australian dairy breeds; approximately 30% purebred Holstein Friesian with the remaining animals predominantly a mixture of Jersey, Australian Red, and Swedish Red animals along with the associated crosses of these various breeds (Verdon *et al.* 2018). Heifer breed was balanced across treatments, allowing each group to represent the genetic variability of the wider milking herd, however given this variability heifer breed was not included in the statistical models. Given the high level of inter-breeding within the herd’s various genetic contributors, we suspect limited effects of breed on the individuals in the present study. Some previous research (e.g. Wagner *et al.* 2015; Neave *et al.* 2022) has, however, included animal breed in models exploring behavioural data, without reporting any breed differences in the results. Future research may find it prudent to explore breed differences in longitudinal effects of varied early-life experiences.

Lastly, CC management differed from –S and +S management in two regards. Firstly, during the pre-weaning period, CC heifers were fed 1 L of milk less per day than pasture-reared +S and –S calves. This discrepancy in fed milk volume arose from differences between commercial farm and experimental calf-rearing protocol. While milk intake can influence physiological development, the present study’s experimental protocol was developed to ensure that pasture-reared calves were fed sufficient volumes to support a hypothesised higher energy consumption resulting from higher activity and thermoregulation needs. While we do not believe this slight difference in daily milk volume would have affected long-term behavioural development, it must be mentioned as a limitation. Previous research has indicated that calves housed on pasture during the pre-weaning stage consume less concentrate than those housed indoors (Noller *et al.* 1959); future research may choose to elucidate differences in feeding behaviour of calves housed in varied environments and how these differences influence longitudinal growth, productivity, and ruminal and behavioural development.

Secondly, +S and –S heifers were weighed weekly through the race adjacent to the stockyards used as the testing arena from the ages of 8–12 weeks, before all animals were weighed in the area monthly from three months onwards. This may have given +S and –S heifers slightly more familiarity with the location compared to CC heifers and may have impacted behaviour. We do, however, suspect that as the vigilance of CC heifers increased only after the introduction of the novel object, this had a negligible effect on results, if any.

### Animal welfare implications

Rearing replacement heifers indoors by hand limits opportunities for these calves to experience maternal and other herd-based social interactions, as well as varied interactions with their environment (Verdon 2021). Such interactions may be necessary for certain developmental processes, influencing outcomes such as fearfulness, grazing behaviour, and cognition. Indeed, the combination of environmental and social stimulation during early life influences brain development in mice (Pietropaolo *et al.* 2004). There is opportunity for management of dairy calves to evolve to better reflect and support the natural behavioural development of the calf, which may, in turn, support the ongoing welfare of the animal (Cantor *et al.* 2019; Whalin *et al.* 2021).

A dairy cow’s behavioural tendencies, influenced by her early-life experiences, have implications for her welfare and productivity, particularly through her long-term adaptability and reactivity (Hedlund & Løvlie 2015). Animals which cope well with social isolation or presentation of a novel object are believed to cope better with the multitude of stressors present in modern dairy systems (Haskell *et al.* 2014). For heifers in the present study, reduced fear of novelty of +S heifers may translate to an improved ability to transition with changes in environment, such as new feeds, or the novel environment of the milking parlour. Future research with the present experimental cohort will aim to confirm this hypothesis, while undertaking similar studies with greater animal numbers and factorial designs in particular would confirm these findings and further elucidate treatment differences.

## Conclusion

Effects of both pasture-rearing and adult contact during the first three months of life are shown to last until at least 18 months of age. The results of the present study suggest that both environmental (pasture-rearing) and social (contact with non-maternal adults) enrichment during early life may have positive effects on artificially reared heifer fearfulness in response to novel stressors, compared to heifers reared in more traditional indoor artificial-rearing systems. The combination of environmental and social enrichment, however, appears to have the strongest effects on heifer development. Future research should explore the combined and discrete effects of environmental and social enrichment on dairy heifer development.

## Supporting information

Field et al. supplementary material Field et al. supplementary material

## Supplementary material

The supplementary material for this article can be found at http://doi.org/10.1017/awf.2024.20.
