# Supplementary material for: Early-life contact with non-maternal adult cows and a pasture-based rearing environment influence behavioural responses of dairy heifers to novelty
Source: Anim Welf. 2024 Apr 8;33:e18. doi: 10.1017/awf.2024.20 (PMC11016361; doi:10.1017/awf.2024.20)
Supplement: Field et al. supplementary material [file S0962728624000204sup001.pdf]

Table S1. Estimates for effects of testing day (1–4) on duration (s) of behaviours observed during a 7-min isolation test. This table shows results of overall tests for the effect of day on behaviour, as calculated with linear mixed effects models (#Equation 1), during a social isolation test. Significant values are denoted by an asterisk and bold type

**Behaviour**	**F (df)**	***P*-value**
Explore environment (s)	2.162 (3, 44)	0.106
Explore gateway (s)	0.383 (3, 41.317)	0.766
Walking (s)	2.866 (3, 44)	**0.047***
Running (s)	2.911 (3, 43)	**0.045***
Vigilance (s)	5.165 (3, 44)	**0.004***
Standing (s)	1.383 (3, 44)	0.261
Grazing (s)	8.454 (3, 44)	**0.000***
Self-grooming (s)	2.510 (3, 44)	0.071

CC = heifers reared commercially in sheds, without adult contact; +S = heifers reared at pasture with adult contact and –S = heifers reared at pasture without adult contact.

Table S2. Estimates for effects of testing day (1–4) on duration (s) of behaviours observed during a 7-min novel object test. This table shows the results of overall tests for the effect of day on behaviour, as calculated with linear mixed effects models (#Equation 1), during a novel object test. Significant values are denoted by an asterisk and bold type

**Behaviour**	**F (df)**	***P*-value**
Explore environment	0.639 (3, 43)	0.594
Walking	3.508 (3, 43)	**0.023***
Running	1.557 (3, 43)	0.214
Vigilance	1,612 (3, 43)	0.201
Standing	5.061 (3, 40.384)	**0.005***
Grazing	6.618 (3, 40.319)	**0.001***

CC = heifers reared commercially in sheds, without adult contact; +S = heifers reared at pasture with adult contact and –S = heifers reared at pasture without adult contact.
